# Biomechanics of Gait during Pregnancy

**DOI:** 10.1155/2014/527940

**Published:** 2014-12-21

**Authors:** Marco Branco, Rita Santos-Rocha, Filomena Vieira

**Affiliations:** ^1^Interdisciplinary Centre for the Study of Human Performance (CIPER), Faculty of Human Kinetics (FMH), University of Lisbon (UL), 1499-002 Cruz Quebrada-Dafundo, Portugal; ^2^Sport Sciences School of Rio Maior (ESDRM), Polytechnic Institute of Santarém (IPS), 2040-413 Rio Maior, Portugal; ^3^Faculty of Human Kinetics (FMH), University of Lisbon (UL), 1499-002 Cruz Quebrada-Dafundo, Portugal

## Abstract

*Introduction*. During pregnancy women experience several changes in the body's physiology, morphology, and hormonal system. These changes may affect the balance and body stability and can cause discomfort and pain. The adaptations of the musculoskeletal system due to morphological changes during pregnancy are not fully understood. Few studies clarify the biomechanical changes of gait that occur during pregnancy and in postpartum period. *Purposes*. The purpose of this review was to analyze the available evidence on the biomechanical adaptations of gait that occur throughout pregnancy and in postpartum period, specifically with regard to the temporal, spatial, kinematic, and kinetic parameters of gait. *Methods*. Three databases were searched and 9 studies with a follow-up design were retrieved for analysis. *Results*. Most studies performed temporal, spatial, and kinematic analysis. Only three studies performed kinetic analysis. *Conclusion*. The adaptation strategies to the anatomical and physiological changes throughout pregnancy are still unclear, particularly in a longitudinal perspective and regarding kinetic parameters.

## 1. Introduction

Pregnancy is a unique time in the life of women with many changes that affect the musculoskeletal system [1]. Over 38 to 42 weeks, women experience several changes in the body's physiology, morphology, and hormonal system. These changes are visible especially in increased weight and skeletal alignment. Other less visible changes are the increased joint laxity and change in the center of gravity. Altogether, these changes affect the balance and body stability and can cause discomfort and pain [2]. The displacement of the center of gravity has been discussed over the years with different statements. Some studies indicate that the center of gravity (CG) moves upward and anteriorly, for example, Foti et al. [3] and Rodacki et al. [4]. Other studies state that the CG shifts on the upper and posterior direction (e.g., Fries and Hellebrandt [5]). Whitcome et al. [6] evaluated the evolution of lumbar lordosis in bipedal hominids, and the results elucidate that the CG moves anteriorly until the fetus reaches 40% of the expected final weight. From that moment, the woman increases the lordotic adjustment which in turn enables the control of the CG, but with greater biomechanical costs [3].

Hormonal changes in women are quite variable throughout pregnancy. However, the hormone relaxin may have a more decisive role in the mechanics of movement, as it provides greater ligament laxity in the pelvis and on the peripheral joints [2, 7, 8]. The concentration peak of relaxin occurs around the 12th week of gestation, which means that there is enough time to act in osteoarticular structures until the end of pregnancy.

One of the aspects that most influences the musculoskeletal system is the increase in maternal weight. When weight is in excess it may cause several adverse maternal effects, including gestational hypertension, gestational diabetes, difficulties during labor, weight retention in postpartum, and subsequent maternal obesity with the risk for unsuccessful breastfeeding [9]. The ideal weight gain during pregnancy is related to the women's weight or body mass index (BMI) before pregnancy. The weight gain may range between 12.5 Kg and 18 Kg for women under weight (BMI < 18.5); between 11.5 Kg and 16 Kg for women with normal weight (18.5 ≤ BMI < 25); between 7 Kg and 11.5 Kg for women who are overweight (25 ≤ BMI < 30); and between 5 Kg and 9 Kg for obese women [9]. The weight gain during pregnancy is related to several factors, such as the amount of blood, the increased volume of the breasts, and the increased fat mass and extracellular fluid [6]. The fetal tissues, the placenta, and the amniotic fluid are related to the weight gain of the fetus [9].

A strategy to control weight gain during pregnancy is by performing structured or recreational physical activity. The guidelines for exercise prescription of the American College of Obstetricians and Gynecologists recommend aerobic exercise consisting of activities that use large muscle groups in a continuous, rhythmic manner [1]. Walking emerges as a highly recommended physical activity for pregnant women. Walking is one of the movements most commonly performed by people in the day-to-day tasks and it is easy to control exercise intensity.

When performing motor tasks such as walking, the adaptations of the musculoskeletal system due to morphological changes during pregnancy are not fully known. Few studies clarify the biomechanical changes of gait that occur during pregnancy and in postpartum.

The purpose of this review was to analyze the available evidence on the biomechanical adaptations of gait that occur throughout pregnancy and in postpartum, specifically with regard to the temporal, spatial, kinematic, and kinetic parameters of gait.

## 2. Methods

### 2.1. Eligibility Criteria

The review was limited to studies meeting the following eligibility criteria: (1) healthy pregnant women with no history of foot, ankle, knee, musculoskeletal or neuromuscular trauma, or disease; (2) women aged between 20 and 40 years; (3) studies performed in the last 14 years; (4) studies performed by means of optoelectronic systems, image analysis, force platforms, and others; (5) outcome measures related to biomechanical variables of gait, including spatial (stride length, step length, and stride width), temporal (single and double support time), kinematic (velocity and cadence), and kinetic (ground reaction forces, joint moment, and joint power) parameters; (6) study design including cross-sectional, follow-up, quasi-experimental trials, and randomized controlled trials.

### 2.2. Information Sources

The studies were identified by searching three databases—ScienceDirect, PubMed, and SciELO—from January 2000 to August 2014. In addition, reference lists of identified studies were also scanned. Studies published in English, Portuguese, Spanish, or French were scanned. Only published full papers were included. Unpublished data, books, conference proceedings, or academic theses were not included.

### 2.3. Search Strategy and Study Selection

The search was performed using the following keywords in English: (1) gait/walking; (2) pregnancy/pregnant; and (3) biomechanics/kinetics/kinematics. These keywords were in first place identified in the titles and in the abstracts. The study selection was conducted in two stages. The first stage was the screening of the titles and abstracts against the inclusion criteria to identify relevant papers. The second stage was the screening of the full papers to identify whether they met the eligibility criteria.

### 2.4. Data Collection Process and Analysis

After screening each paper, the following data were extracted: (1) study design; (2) sample size; (3) age of the women; (4) gestational phase of data collection; (5) outcome variables: spatial variables, temporal variables, kinematic variables, and kinetic variables; (6) biomechanical instruments. The studies were, therefore, described according to these characteristics and outcome measures.

## 3. Results

### 3.1. Study Selection and Characteristics

The search yielded 741 articles from the three databases: ScienceDirect (711), PubMed (28), and SciELO (2). After removing duplicate articles 21 articles were considered for analysis. Articles were then screened on the basis of title and abstract, with 16 excluded. After searching the reference lists of the selected articles, 4 more articles were included. The remaining 9 full papers were examined in detail, found to meet the inclusion criteria, and then included in the review.

The characteristics of the studies included in the review are presented in Table 1. All studies have follow-up design. The sample sizes across the studies ranged from 2 [9] to 124 [7]. All studies reported the mean age and standard deviation, except one [10]. Different studies used video-based systems for kinematic analysis, except for two studies that use optoelectronic systems [11, 12] and one that only uses pedometers [7] to analyze the temporal and spatial parameters. Only three studies report a kinetic analysis [10, 12, 13] and most studies provide an analysis of the kinematic, spatial, and temporal parameters.

### 3.2. Outcome Measures: Spatial and Temporal Parameters

The variables of stride length, step length, stride width, base of support, and single and double support time are the spatial and temporal parameters analyzed by the papers reviewed. Most papers reviewed showed changes in spatial and temporal parameters in late pregnancy, specifically, a significant decrease in the length of the gait cycle [11, 13–15], and in step length [11–14], and a significant increase of the double support time [3, 11, 14]. Other studies showed a significant reduction in the single support time [3, 14] and a significant increase in step width [10, 15]. Forczek and Staszkiewicz [12] also found a significant increase of the base of support. Nevertheless, the remaining studies suggest that pregnant women have the need to increase the body stability and use the parameters listed above to meet those demands.

### 3.3. Outcome Measures: Joint Kinematics

The variables of velocity (or speed) and cadence are the kinematic parameters analyzed by the studies included in the review. Most papers reviewed showed changes in kinematic parameters in late pregnancy, specifically, a significant decrease in speed [12, 13, 16] and a significant reduction in the gait cadence [12, 16].

The joint kinematics of the lower limb shows few changes throughout pregnancy. The angular displacement of the pelvis increases in the anterior tilt of approximately 5 degrees [3, 13]. The joints of the lower limb in the sagittal plane show an increase in hip flexion during stance phase [3, 11, 13], an increase of knee flexion during the terminal stance phase [14], a decrease of knee extension [11], and a decrease of ankle dorsiflexion and plantarflexion [11, 13]. In the frontal plane, Gilleard [15] found a reduction in the amplitude of the unilateral elevation of the pelvis. The hip joint had different results considering the two studies performed: Foti et al. [3] found a peak with greater magnitude in the hip adduction; Branco et al. [11] found a decrease of this peak.

### 3.4. Outcome Measures: Joint Kinetics

Few studies have evaluated the kinetic parameters of gait during pregnancy [10, 12, 13]. Foti et al. [3] analyzed the joint moments and joint powers with and without normalizing the weight of the women in late pregnancy. Although they found several changes in these parameters without normalization, the authors recommend the analysis with normalization [13]. The analyses performed with normalized data found a significant increase in the hip extensors moment [17] and a significant decrease in the knee extensors moment [17] and in the ankle plantarflexors moment [3, 17] in the sagittal plane. In the frontal plane, there was an increase of the hip abductors moment [3] and in the knee adductors moment [17].

## 4. Discussion

The purpose of this review was to analyze the available evidence on the biomechanical adaptations of gait that occur throughout pregnancy and in postpartum. Data from 9 follow-up studies were included in the analysis.

Women are subjected to various anatomical and physiological changes throughout pregnancy. However, the adaptation strategies that are pursued are still unclear [12]. Two of the situations they face are, on the one hand, the reduction of energy costs associated with gait and, on the other hand, maximizing safety during the motor task. The later strategy seems to be dominant because the increase in step width is associated with a greater energy cost during walking [18]. This parameter has great consistency between studies. In addition, the development of pregnancy is associated with a reduction in stride length, which together allow the woman to have a larger base of support [12]. Another way found by pregnant women to ensure greater body stability is keeping their feet on the ground for a longer time during the gait cycle and decreasing the single support time, which seems to be also a strategy to support the increased weight [3]. In fact, the medial-lateral instability of the women as the pregnancy progresses is one result that seems to have great importance among different types of biomechanical parameters. However, so far the medial-lateral component of the ground reaction forces shows no significant changes but tends to be higher in late pregnancy [10]. This observation requires further research.

Many of the biomechanical changes occur in the pelvis or in adjacent joints. Although there are few kinematic changes in late pregnancy, most of them are related to the angular motion of the pelvis and the hip joint. The result with greater emphasis between studies is the anterior tilt of the pelvis. The position taken by the pelvis in late pregnancy seems to be a consequence of the weight of the uterus, placenta, and fetus placed on the anterior zone of the body and the weaker capacity to produce force by the rectus abdominis. However, the function of this anterior tilt and the increase of the lumbar lordosis are related to the maintenance of the trunk in an upright position [3]. The decrease in the range of motion of the pelvis in the frontal and transverse planes suggests that this may be a way to control the angular momentum caused by the increase of the moment of inertia of the trunk in late pregnancy [15]. These changes bring consequences for the muscles attached to the pelvis, particularly a greater participation of the abductors and extensors muscles of the hip, which, combined with a higher stretch derived from the anterior tilt of the pelvis, will contribute to lower back, pelvis, hip, and sacroiliac pain [3, 17]. These types of pain are also associated with a decrease in the participation of the knee extensors and ankle plantarflexors [17]. The changes in angular motion of the ankle may also be associated with other issues of pregnant women, including a decrease of dorsiflexion that is related to pain and higher probability to trip and fall [13].

*Limitations.* The strength of this review is that a systematic methodology was adopted to identify relevant studies on the biomechanics of gait during pregnancy. However, the review has some limitations. Articles published in languages other than those mentioned above may have been excluded. The search was performed only in peer-reviewed published articles since 2000. There is a possibility of missing data published previously or other unpublished data that were not included in the analysis. The small sample sizes and the different periods of data collection may have introduced some bias in the analysis performed. The lack of studies about this subject as well as the consistency of the outcome measures is a major limitation to perform a meta-analysis. Cautions should be taken when drawing conclusions about gait adaptions throughout pregnancy, due to the limitations mentioned above.

## 5. Conclusions

The evidence from this review suggests biomechanical adaptations of gait throughout pregnancy. Further research is required using common outcome measures and standard follow-up periods of data collection (weeks of gestation and postpartum period). Most of the results presented in this review are consistent between studies. However, there is a great scarcity of studies addressing the gait biomechanics of pregnant women in a longitudinal perspective. There is a need to examine closely the kinematics of the woman while walking considering the beginning of pregnancy, in order to confirm the influence of morphological changes in the angular motion of the lower limb segments during the course of pregnancy.

Very few studies analyzed the kinetics of gait of pregnant women. Nevertheless, these data are seen as essential to understand the magnitude and implications of changes in the welfare of women. In future research, there must be a special focus on the analysis of moments and joint powers to understand the changes in muscle participation and if there are changes in the type of contraction during motor tasks.

It is not possible to understand the influence of morphological changes, if body composition and anthropometric variables are not quantified throughout pregnancy. Further research is required to understand to which extent do these variables influence the biomechanical parameters.

No studies were found addressing the effects of physical activity, low back, and pelvic pain or maternal weight control on the gait biomechanical adaptations of gait throughout pregnancy and postpartum.

## Figures and Tables

**Table 1:** Studies included in the review, listed by chronological order of publication, regarding authors, sample size, data collection phases, sample mean age, outcome variables, and biomechanical instruments used.

Authors	Sample size (*N*)	Data collection phase(s)	Sample mean age (years)	Outcome variables	Biomechanical instruments
Foti et al. (2000) [3]	2, before pregnancy 15, 3rd trimester 13, postpartum	3rd trimester 1 year postpartum	32	Time and distance Kinematic Kinetic	60 Hz video cameras 1 AMTI force platform

Huang et al. (2002) [17]	10, experimental group 10, control group	1st trimester 2nd trimester 3rd trimester	⋯	Joint moments	EVA motion analysis system

Lymbery and Gilleard (2005) [10]	13	3rd trimester 8 weeks postpartum	27.8 ± 1.2	Spatial Temporal Ground reaction forces	60 Hz EVA motion analysis system 1 Kistler force platform

Carpes et al. (2008) [14]	7	2nd trimester 3rd trimester Until 4th month postpartum	23 to 35	Spatial Temporal 3D kinematics	60 Hz video cameras

Falola et al. (2009) [16]	124	From 2nd to 9th months of gestation	26 ± 4.6	Spatial Temporal	Pedometers Dista F100 Basic

Hagan and Wong (2010) [13]	2	Before pregnancy 1st trimester 2nd trimester 3rd trimester 12 to 16 weeks postpartum	21 and 39	Spatial Temporal 2D kinematics	30 Hz video cameras

Forczek and Staszkiewicz (2012) [12]	13	Before 3rd trimester Half year postpartum	29.15 ± 3.5	Time and distance ROM Base of support	Vicon 250 3D system

Branco et al. (2013) [11]	22	2nd and 3rd trimesters	32.5 ± 2.6	Spatial Temporal 3D kinematics	200 Hz Qualisys Oqus 300

Gilleard (2013) [15]	9	18, 24, 32, and 38 weeks 8 weeks postpartum	32.5 ± 4.3	Spatial Temporal Trunk kinematics	60 Hz EVA motion analysis system 1 Kistler force platform

2D: two-dimensional analysis; 3D: three-dimensional analysis; ROM: range of motion.
